# What can be observed in intervertebral cartilage endplate with aging? An animal model study of excessive axial mechanical loading

**DOI:** 10.3389/fmed.2024.1429208

**Published:** 2024-11-05

**Authors:** Zhouyang Hu, Fan He, Xinhua Li, Bei Jiang, Shuaifeng Yan, Jun Tan, Lijun Li

**Affiliations:** ^1^Huazhong University School of Science and Technology Union Shenzhen Hospital, Shenzhen, China; ^2^Department of Spine Surgery, Shanghai East Hospital, School of Medicine, Tongji University, Shanghai, China; ^3^Department of Spine Surgery, Shanghai Jiaotong University First People's Hospital, Shanghai, China

**Keywords:** cartilage endplate degeneration, rat model, axial controllable loading, Schmorl’s node, Ilizarov external fixator

## Abstract

**Introduction:**

The cartilage endplate (CEP) plays a crucial role as both a mechanical barrier and nutrient channel for the intervertebral disc, but it is vulnerable to excessive axial loading. We modified the Ilizarov external fixator and applied it to the CEP of the rat tail to impose diurnal, controllable excess axial loading. The objective was to measure morphological changes in the CEP when subjected to loading during the aging process.

**Methods:**

Two Kirschner wires were, respectively, inserted into the center of the eighth and ninth coccygeal vertebrae (Co8/9) of rat (*n* = 54) to apply axial loading to the CEP. A remote control device was used to establish the diurnal loading schedule. At the end of 4, 8, and 12-week periods, the Co8/9 CEPs in each group were analyzed using MRI, histological staining, and immunohistochemical staining techniques.

**Results:**

The novel Ilizarov model that we modified successfully induced degeneration of the rat coccygeal CEP. MRI analysis revealed significant degenerative changes in the loaded Co8/9 CEP, including decreased signal intensity and the formation of Schmorl’s nodes at 8 and 12 weeks. Histological examination showed progressive CEP degeneration (CEPD), characterized by decreased microporosity, thinning, and structural irregularities. Immunohistochemical analysis demonstrated a significant reduction in Aggrecan and Collagen II expression in the CEP and nucleus pulposus over time. Control and sham groups maintained normal CEP structure and composition throughout the study period.

**Conclusion:**

Excessive axial loading induced CEPD in the rat tail, primarily characterized by the formation of Schmorl’s nodes and a reduction in CEP microporosity in this study. Our modified Ilizarov rat tail compression model, featuring stable and controllable axial loading capabilities, provided an alternative experimental paradigm for further investigation into CEPD.

## 1 Introduction

The cartilage endplate (CEP) plays a crucial role in the intervertebral disc serving as a connection between the upper and lower vertebral bodies, the annulus fibrosus, and the nucleus pulposus. Functioning as a central biomechanical barrier, CEP is responsible for absorbing and dispersing various types of mechanical forces from the trunk. Additionally, its micro-porous structure functions as a vital channel for nutrient supply from vertebrae to the gel-like nucleus pulposus (1). When the CEP undergoes degeneration, it has significant implications for the intervertebral disc. This degeneration disrupts the supply of nutrients to the surrounding nucleus pulposus cells and creates abnormal intramedullary pressure within their cellular micro-environment. These factors can eventually lead to impairments and even breakdown of the nucleus pulposus cells (2, 3). While CEP is vulnerable to various types of forces, excessive axial load is a crucial factor in the development of CEP degeneration (CEPD) (4, 5). CEP is especially prone to structural failure under excessive axial compression. This failure is key in triggering the onset of degenerative disc disease (6).

A comprehensive understanding of the underlying pathophysiology of CEPD is valuable, and appropriate animal models are essential for investigating CEPD and developing potential therapeutic interventions. Over the past decades, numerous productive efforts have been made for creating an effective CEPD animal model, ranging from osteoporosis modeling, chemicals injection as well as gene knockout techniques. A recent systematic review has highlighted the significant application value of vertebrate models in studying intervertebral disc degeneration caused by CEP injury. The review asserted that the cartilage endplate structure in vertebrates more closely resembles that of humans compared to invertebrates. Small vertebrate models offer distinct advantages, including shorter experimental cycles and higher repeatability, making them particularly suitable for investigating pathological and physiological changes associated with CEP damage. These models provide crucial insights into the mechanisms underlying CEP damage-induced intervertebral disc degeneration and inform the development of related treatment strategies (7). However, there has been a lack of exploration regarding *in vivo* biomechanical injuring approaches (8–10). Nevertheless, the classic biomechanical apparatus Ilizarov-type external fixator has been successfully employed and modified not only in animal intervertebral disc degeneration study but also in the field of orthopedic surgery such as deformity reconstruction and fracture management (11–13). However, prominent disadvantages of this apparatus were proposed as unchanged loading mode and imprecise loading output during long-term performance. To the best of our knowledge, no prior studies have investigated the specific impact of precise excessive axial loading on CEPD onset.

Therefore, in the current study we refined the Ilizarov apparatus with a stable loading supply and set a novel diurnal-loading mode to induce CEPD in rat tail disc. Based on the above perspectives, we aimed to observe the morphological pathology of CEP during various stages of degeneration under excessive axial loading.

## 2 Methods

### 2.1 Ethical approval and animal grouping

The experimental protocols were reviewed and approved by the Ethics Committee on Animal Studies of Shanghai East Hospital, Tongji University School of Medicine. Male Sprague–Dawley rats (3 months old) were used in this study (SLAC Laboratory Animal Co., Ltd., Shanghai, China). All rats were group-housed in standard plastic cages by 3 per cage and raised in the specific pathogen-free (SPF) laboratory. A total of 54 rats were randomly assigned into three groups: the operational group (*n* = 18), the sham group (*n* = 18), and the control group (*n* = 18), respectively. In the operational group, Kircher wires were inserted into eighth and ninth coccygeal vertebrae (Co 8/9), followed by loading (detailed procedure in section 2.2). In the sham group, the Kircher wires were inserted without performing loading. The control group served as a blank control without intervention. Six rats from each group were randomly sacrificed at 4, 8 and 12 weeks for analysis, respectively.

### 2.2 Compression device establishment

**Figure 1:**
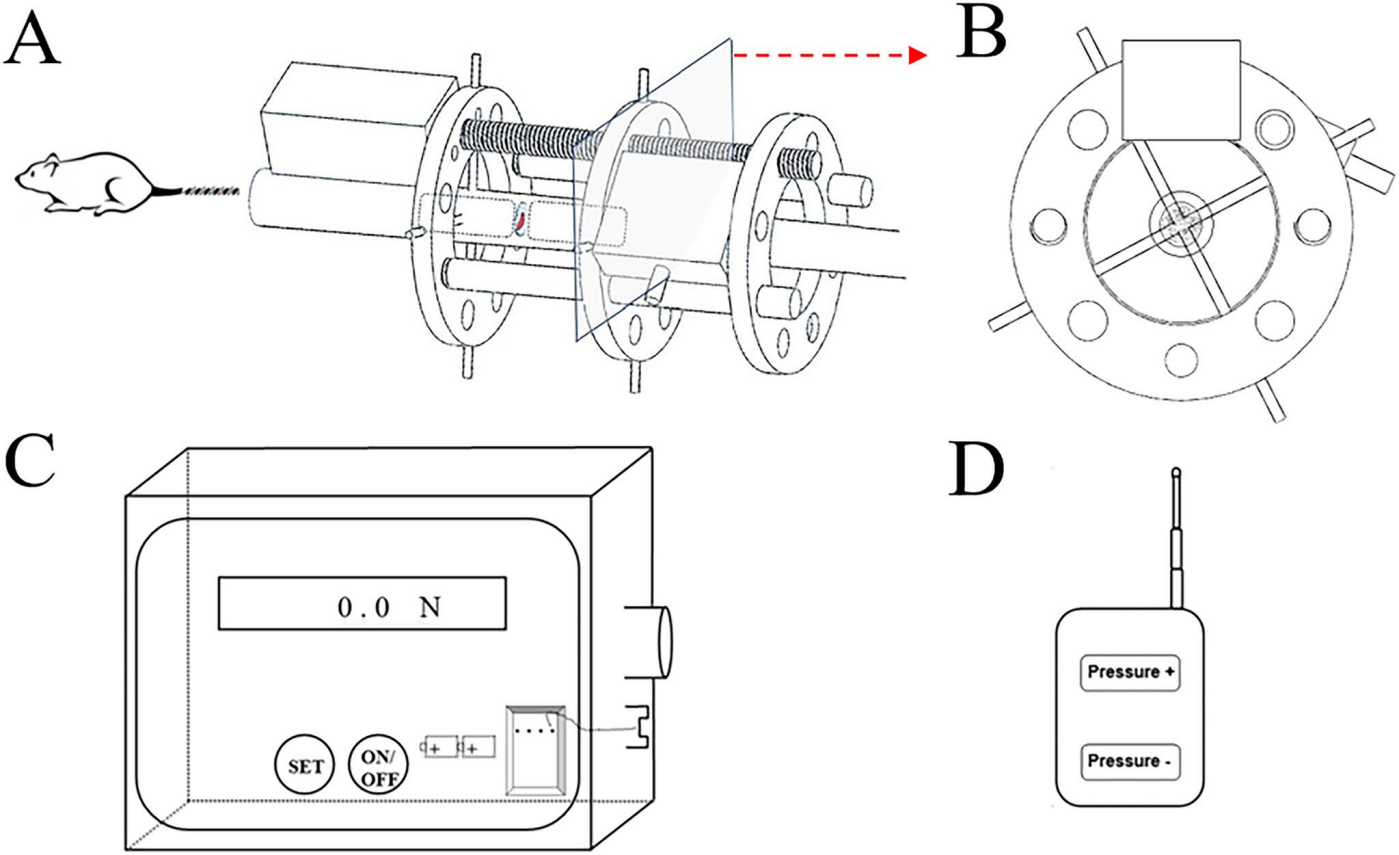
Constructive schematic of the custom-build compressive loading device. The compressive unit with **(A)** frontal view; **(B)** axial view; **(C)** the control box with signal receiver and power bank; **(D)** the infrared remote controller.

The custom-made device comprises three main components (Figure 1):

- Compressive unit: a geared motor with a screw rod penetrating three aluminum rings (with drilled holes); a pressure sensor inserted between two aluminum rings (Figures 1A,B).
- Control unit: connects the compressive unit and displays real-time compressive force (Figure 1C).
- Infrared remote controller: allows adjustment of loading parameters (Figure 1D).

### 2.3 Animal modeling

**Figure 2:**
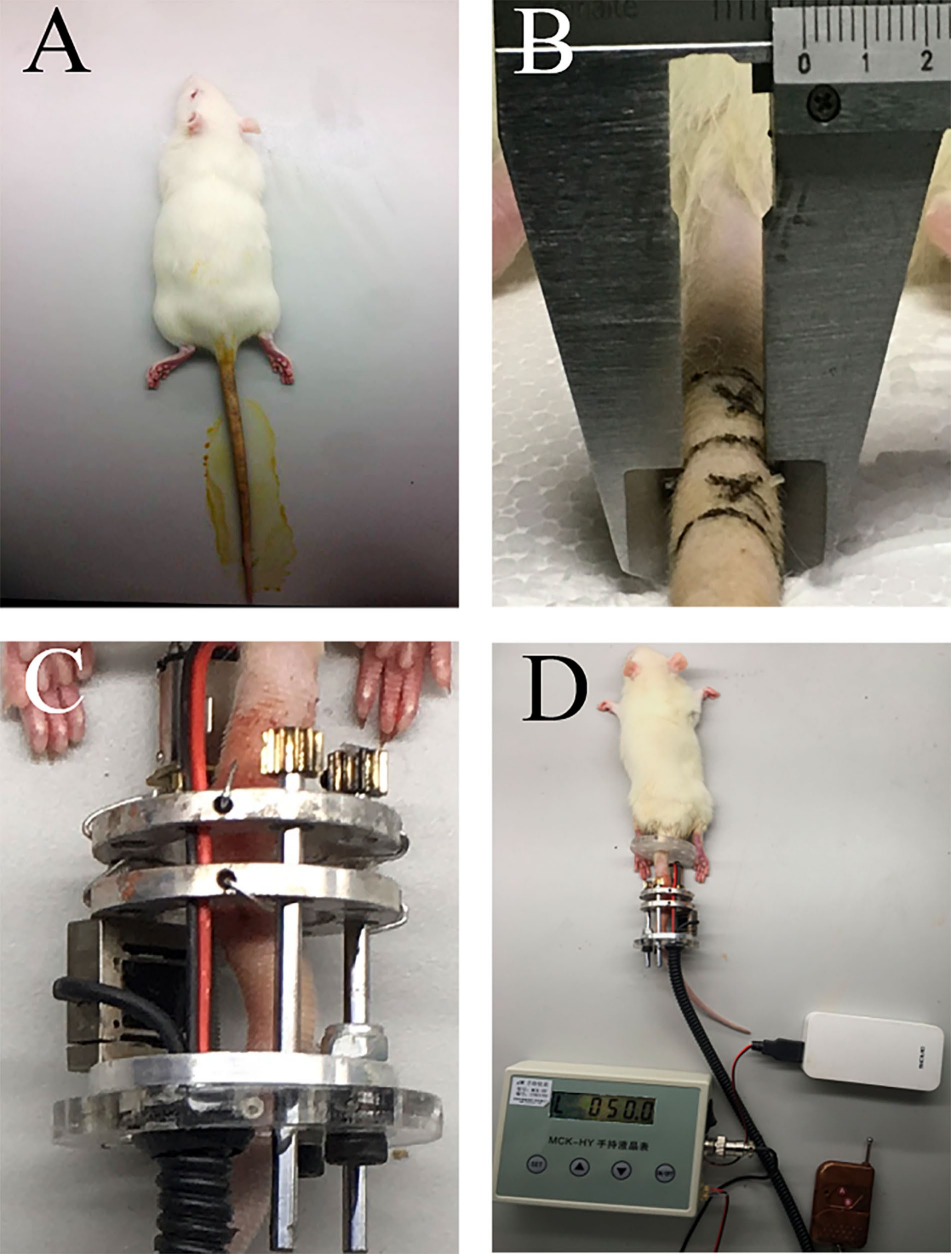
The schematic of installation. **(A)** anesthesia and disinfection; **(B)** diameter measurement of the Co 8/9 disc, the two cross marks indicate the puncture points; **(C)** the loading status of the Co 8/9 disc; **(D)** the general situation after installation.

As shown in Figure 2, the model was both installed in the tail of Sprague–Dawley rats in the operational group and B. Animals were anesthetized with the injection of 4% chloral hydrate (0.1 mL/kg body weight, Sangon Biotech, Shanghai, China) via intraperitoneal injection (Figure 2A). A prone position was taken for the operation. First, the compressive unit was penetrated through the tail. Two Kirschner wires (0.9 mm) were, respectively, inserted into the center of Co 8/9through two aluminum rings. Next, the loading command was issued via the infrared remote controller to apply pressure, causing extrusion of the disc between the Co8 and 9 vertebrae in the axial direction. As previous studies proposed (14, 15), a compressive load of 1.3 MPa was applied to the targeted Co 8/9 disc. The diameter and area of the Co 8/9 disc were measured and calculated using a Vernier caliper tightened to the skin of the Co 8/9 disc (Figure 2B). Then, the loading value required was calculated according to the pressure-force-area formula. Loading was performed continuously until the determined value [in Newtons (N)] was reached, which was displayed in real-time on the control box screen (Figures 2C,D). The loading regimen was set to 8 h of loading and 16 h of unloading daily, and repeated for 4, 8, and 12 weeks in the operational group (16). The sham group received no loading after wire insertion. The control group was set as a blank control without any operation. For all animals in Groups A, B and C, amoxicillin trihydrate was subcutaneously injected (7 mg/kg body weight, Yuanye Bio-Technology Co., Ltd., Shanghai, China) to prevent infection within 1 week after installation. Analgesic ibuprofen granules (5 mg/kg body weight, HPGC, Harbin, China) were administered intragastrically throughout the experiment for animal welfare for all animals in across all three groups.

### 2.4 Magnetic resonance imaging examination

Six rats were randomly selected from each group at each timepoint (4, 8 and 12 week). Animals were one-by-one anesthetized with the injection of 4% chloral hydrate (0.1 mL/kg body weight, Sangon Biotech, Shanghai, China) via intraperitoneal injection. Then, each rat was sent for MRI examination. An MRI scanner (Achieva 3.0 T; Philips Medical Systems, Best, the Netherlands) was used with the following parameters: T1-weighted imaging: TR = 300 ms, TE = 20 ms, FOV = 60 mm × 60 mm, data matrix = 148 × 150, and slice thickness = 1 mm. T2-weighted imaging: TR = 5,000 ms, TE = 50 ms, FOV = 60 mm × 60 mm, data matrix = 200 × 200, and slice thickness = 1 mm. The modified Outerbridge Score (17), which is a five-point scale was used for assessing the grade of CEPD. A score of 0 represents healthy, normal cartilage, while a score of 4 represents the most severe degeneration, where the subchondral bone is exposed due to severe loss of the cartilage layer. Two experienced radiologists took part as independent observers. After MRI, 6 rats at each timepoint were sacrificed and their Co8/9 discs were then dissected for the following histological and immunohistochemical staining.

### 2.5 Formalin-fixed paraffin-embedded tissue preparation

At each timepoint, 6 rats in three groups were, respectively, sacrificed by fixation procedure using 4% paraformaldehyde perfused via the circulation system (18). The targeted Co8/9-disc unit (containing complete superior and inferior CEP) was extracted and fixed in 4% paraformaldehyde, decalcified in 10% 0.5 M ethylenediaminetetraacetic acid (EDTA; Servicebio Co., Ltd., Wuhan, China) and then embedded in EM-400 embedding medium paraffin. Disc tissues were sectioned at 5 μm thickness using a microtome (Leica RM2235, Biosystems, Wetzlar, Germany).

### 2.6 Histological evaluations

The Co8/9-disc unit sections of 6 rats at each timepoint were stained with hematoxylin and eosin (H&E) and analyzed qualitatively for observing morphological changes of degenerated CEP using a microscope (Leica DM6000B, Microsystems, Wetzlar, Germany). Three sections were prepared from each Co8/9-disc unit. A histological classification system was used for assessing the CEP morphological pathology (19). The system includes evaluating the cartilage endplate, annulus fibrosus, and nucleus pulposus, classified into six subcategories for scoring. Each item is graded from 0 to 2, with a total score ranging from 0 (healthy) to 12 (degenerated).

### 2.7 Immunohistochemical staining

Collagen II and Aggrecan were evaluated as potential markers for CEPD. Immunohistochemical (IHC) staining was conducted using the following primary antibodies: mouse monoclonal Collagen II antibody (1:150, ab185430, Abcam, Cambridge, United Kingdom) and mouse monoclonal Aggrecan antibody (1:150, ab3773, Abcam, Cambridge, United Kingdom). Detection was achieved using 3,30-diaminobenzidine (DAB), resulting in positive cells being stained brown, while nuclei were counter-stained blue with hematoxylin. All stained specimens were captured under a microscope (Leica DM6000B, Microsystems, Wetzlar, Germany), and subsequent semi-quantitative analysis was performed using Image-Pro Plus 7.0 software (Media Cybernetics, Inc., Rockville, United States). The average optical density was measured from images at 400× magnification.

### 2.8 Statistical analysis

The data were presented as mean ± standard deviation (SD). Cohen’s weighted kappa was used to measure agreement between two radiologists when evaluating the modified Outerbridge Score of CEP MRI scans. The weighted kappa coefficient and 95% confidence interval (CI) were calculated. Analysis of variance (ANOVA) was performed for determining significant differences of histological grading scores, as well as the average optical density of Aggrecan and Collagen II, among three groups (three groups were compared pairwise to each other at each time point). The level of statistical significance was set at *p* < 0.05.

## 3 Results

### 3.1 Animals

In the operational group, one rat failed to maintain the loading device due to necrotic tail end loss after 4 weeks of loading. In the sham group, 1 rat died of the anesthesia intolerance during the operation. The remaining animals survived throughout the experimental period.

### 3.2 MRI findings

**Figure 3:**
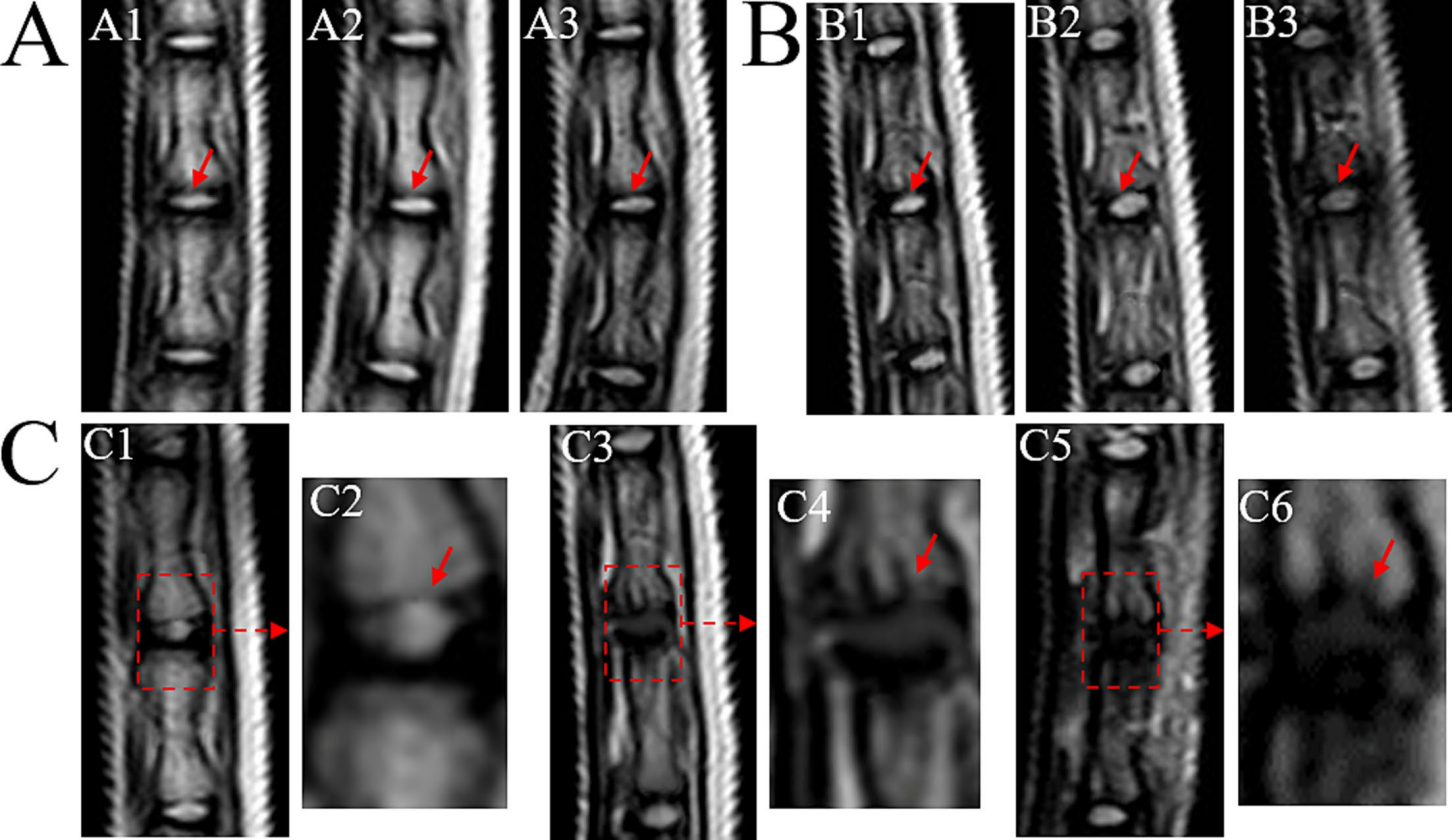
MRI T2 images of loaded Co8/9 intervertebral disc. **(A)** the control group; **(B)** the sham group and **(C)** the loading group after 4, 8 and 12 weeks of operation. A1/B1/C1/C2 indicate MRI performed after 4 weeks, A2/B2/C3/C4 indicate MRI performed after 8 weeks and A3/B3/C5/C6 indicate MRI performed after 12 weeks. The red dotted arrows indicate the punctured Co8/9 unit, the red solid arrows indicate the cartilage endplate.

The control group exhibited consistent high signal intensity in T2 images of the Co 8/9 disc, with clear CEP nucleus pulposus boundaries and normal intervertebral disc height (Figure 3A). The sham group showed no significant signal changes in the Co 8/9 disc at 4, 8, and 12 weeks, with intact CEP and no lesions when comparing with the control group (Figure 3B1 vs. Figure 3A1; Figure 3B2 vs. Figure 3A2; Figure 3B3 vs. Figure 3A3). The operational group demonstrated significant signal decrease in the Co 8/9 disc when compared to the sham and control group at each timepoint (Figures 3C1,C2 vs. Figures 3A1,B1; Figures 3C4,C5 vs. Figures 3A2,B2; Figures 3C5,C6 vs. Figures 3A3,B3). Although the general shape of the CEP appeared largely unchanged after 4 weeks of loading, pronounced lesions in the CEP and subchondral bone emerged at 8 and 12 weeks. As depicted in Figures 3C4,C6, disc height significantly decreased, and notable herniations (Schmorl’s nodes) were observed. Table 1 showed the grading scores of the loaded Co8/9 CEP in three groups by using the modified Outerbridge Score system. Inter-rater reliability between the two radiologists was high (weighted kappa = 0.946, 95% CI: 0.895–0.998, *p* < 0.001).

**Table 1:** The grading scores of Co8/9 CEPs in three groups at the end of 4, 8, and 12-week periods.

		4 weeks					8 weeks					12 weeks				
Modified outerbridge scores		0	I	II	III	IV	0	I	II	III	IV	0	I	II	III	IV
Radiologist 1	Operation group	0	2/6	2/6	1/6	0	0	0	0	4/6	2/6	0	0	0	0	6/6
	Sham group	4/6	1/6	0	0	0	6/6	0	0	0	0	5/6	1/6	0	0	0
	Control group	6/6	0	0	0	0	6/6	0	0	0	0	6/6	0	0	0	0
Radiologist 2	Operation group	0	3/6	1/6	1/6	0	0	0	0	3/6	3/6	0	0	0	0	6/6
	Sham group	4/6	1/6	0	0	0	5/6	1/6	0	0	0	6/6	0	0	0	0
	Control group	6/6	0	0	0	0	6/6	0	0	0	0	6/6	0	0	0	0

### 3.3 HE staining evaluation

**Figure 4:**
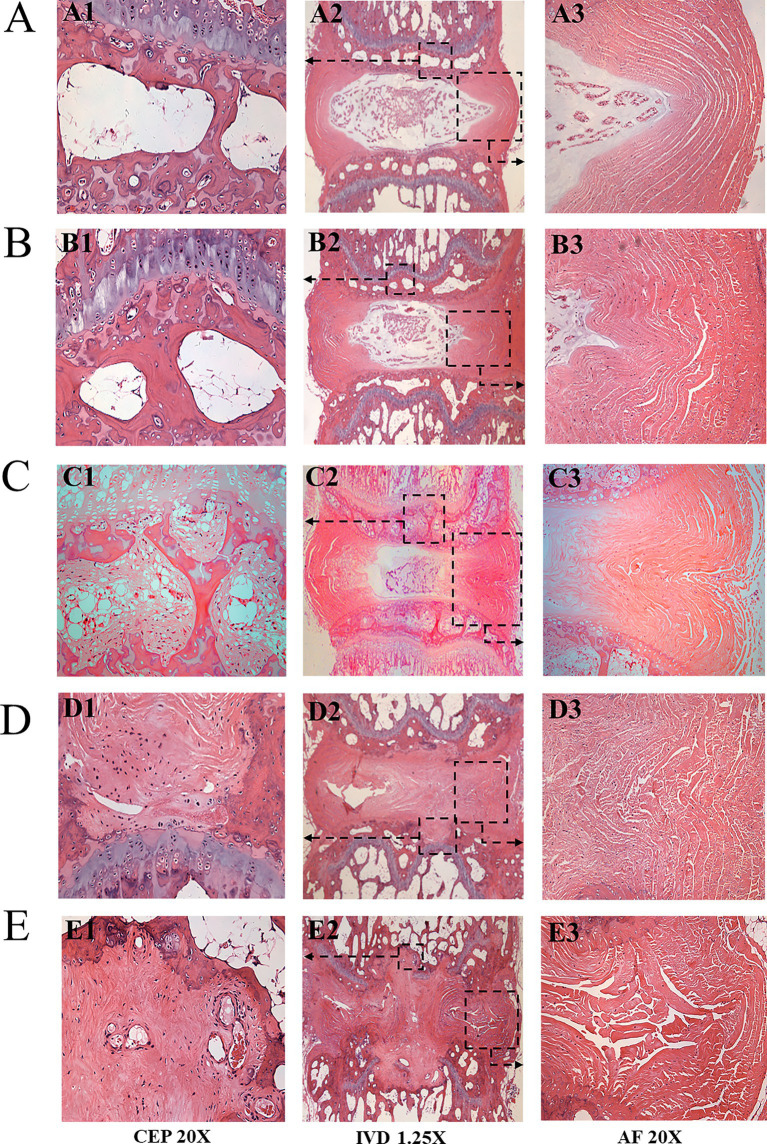
HE stainings of the loaded Co8-9 intervertebral disc unit in three groups. **(A)** the control group at 12 weeks; **(B)** the sham group at 12 weeks; **(C)** 4 weeks after operation; **(D)** 8 weeks after operation; **(E)** 12 weeks after operation. (A1/B1/C1/D1/E1 indicated the Co8/9 CEP; A2/B2/C2/D2/E2 indicate Co8/9 intervertebral disc unit; A3/B3/C3/D3/E3 indicated annulus fibrosus; the black arrow shown the typical Schmorl’s nodule, CEP, cartilage endplate; AF, annulus fibrosus).

In the control group, the CEP exhibited a consistent and uniform structure with normal micro-porosity distribution. Nucleus pulposus cells displayed rounded morphology and no cell clusters or fibrosis were observed. The AF ring demonstrated well-organized and lamellar architecture with clear CEP, annulus fibrosus and nucleus pulposus boundaries (as depicted in Figure 4, control group A1–A3).

In the sham group, the CEP structure remained normal and intact. However, a curved annulus fibrosus structure was noted (Figures 4B1–B3). Histological scores following 4, 8 and12 weeks of loading were 0.4 ± 0.8, 0.7 ± 0.6, and 1.1 ± 0.5, respectively (as summarized in Table 2).

**Table 2:** The histological grading scores of the Co8/9 disc in three groups ( $\bar{X}$ + S).

	4 weeks	8 weeks	12 weeks
Operational group	5.4 ± 2.1*	7.6 ± 2.5*	11.3 ± 1.2*
Sham group	0.4 ± 0.8	0.7 ± 0.6	1.1 ± 0.5
Control group	0	0	0

*indicates the statistical differences compared to Group B (*p* = 0.02, 0.01 and 0.00, respectively).

In the operational group:

1. After 4 weeks’ loading, in the degenerated CEP, inflammatory cells accumulated within the micro-porosity region. At the same time, the diameter of the micro-porosity increased (Figures 4C1,C2); the size and the water content of the nucleus pulposus decreased (Figure 4C3). The annulus fibrosus was compressed into an S-type cycle under loading (Figure 4C3). The histological score averaged 5.4 ± 2.1 (Table 2);
2. After 8 weeks’ loading, a significant thinned CEP was shown. The number of micro-porosities in CEP decreased and its structure became irregular. The calcified nucleus pulposus ruptured through the superior and inferior endplates (Schmorl’s node) as shown in Figures 4D1,D2; The annulus fibrosus was severely ruptured and disordered, as shown in Figure 4D3. The histological score averaged 7.6 ± 2.5 (Table 2); and
3. After 12 weeks’ loading, the micro-porosities in the CEP almost disappeared, and the structure of CEP was severely broken. The notochordal cells almost disappeared, and a large amount of cartilage fibrosis and calcification occurred in the central region. Increased nucleus pulposus tissues penetrated vertebral area, and annulus fibrosus was severely ruptured and disordered (Figures 4E1–E3). The histological score averaged 11.3 ± 1.2. As shown in Table 2, statistical analysis revealed significant differences between Group A and Group B at all timepoints (*p* = 0.02, 0.01 and 0.00 at timepoint of 4, 8 and 12 weeks, respectively) The rats in the control group did not undergo any surgical procedure, and histological examination revealed no signs of degeneration.

### 3.4 Immunohistochemical analysis

**Figure 5:**
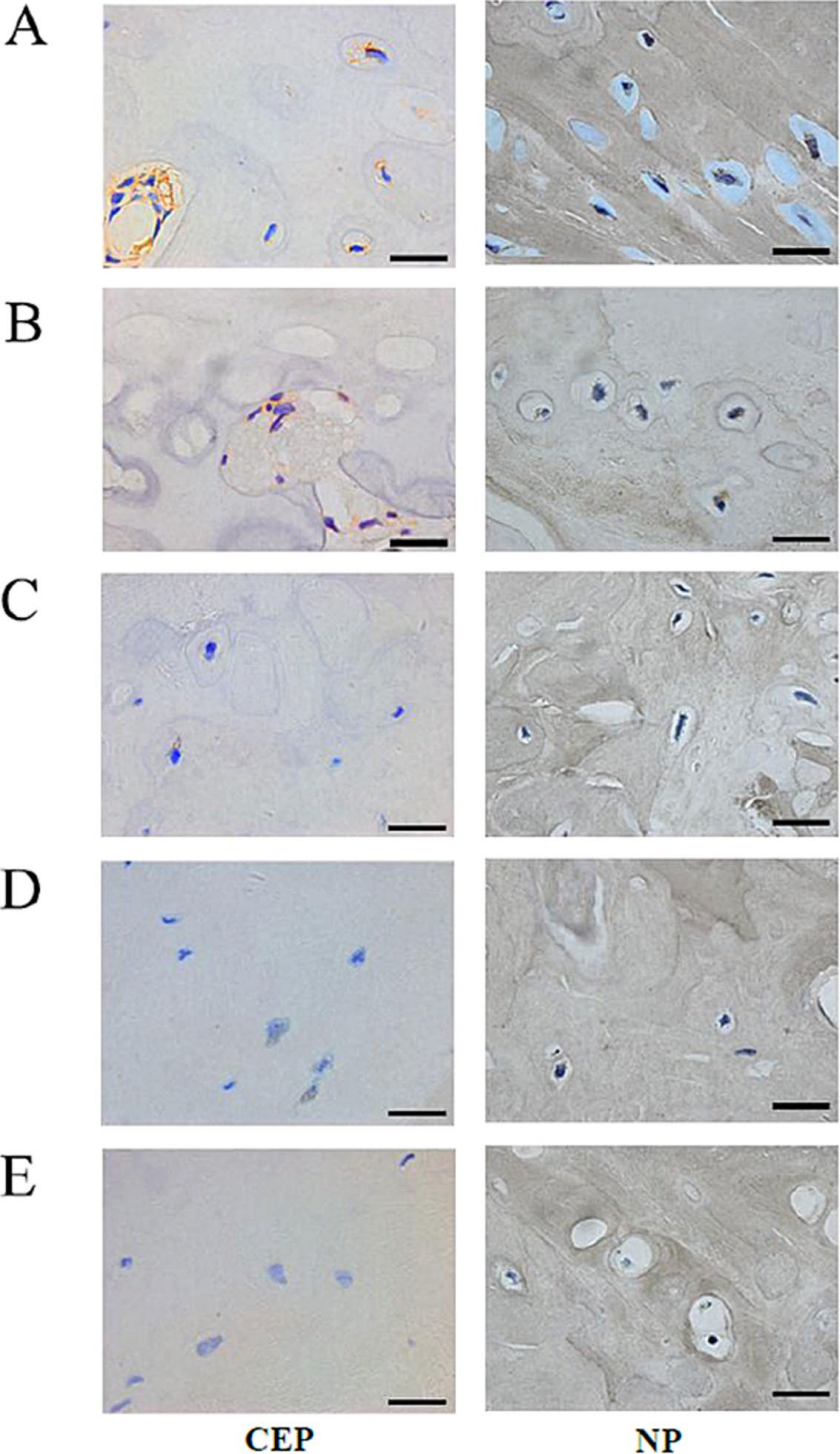
Immunohistochemical staining of the matrix *Aggrecan* and *Collagen II* of Co8/9 intervertebral cartilage endplates and nucleus pulposus cells in three groups. **(A)** the control group at 12 weeks; **(B)** the sham group at 12 weeks; **(C)** the operational group after loading of 4; **(D)** 8 and **(E)** 12 weeks. (Bar = 20 μm, CEP, cartilage endplate; NP, nucleus pulposus).

**Figure 6:**
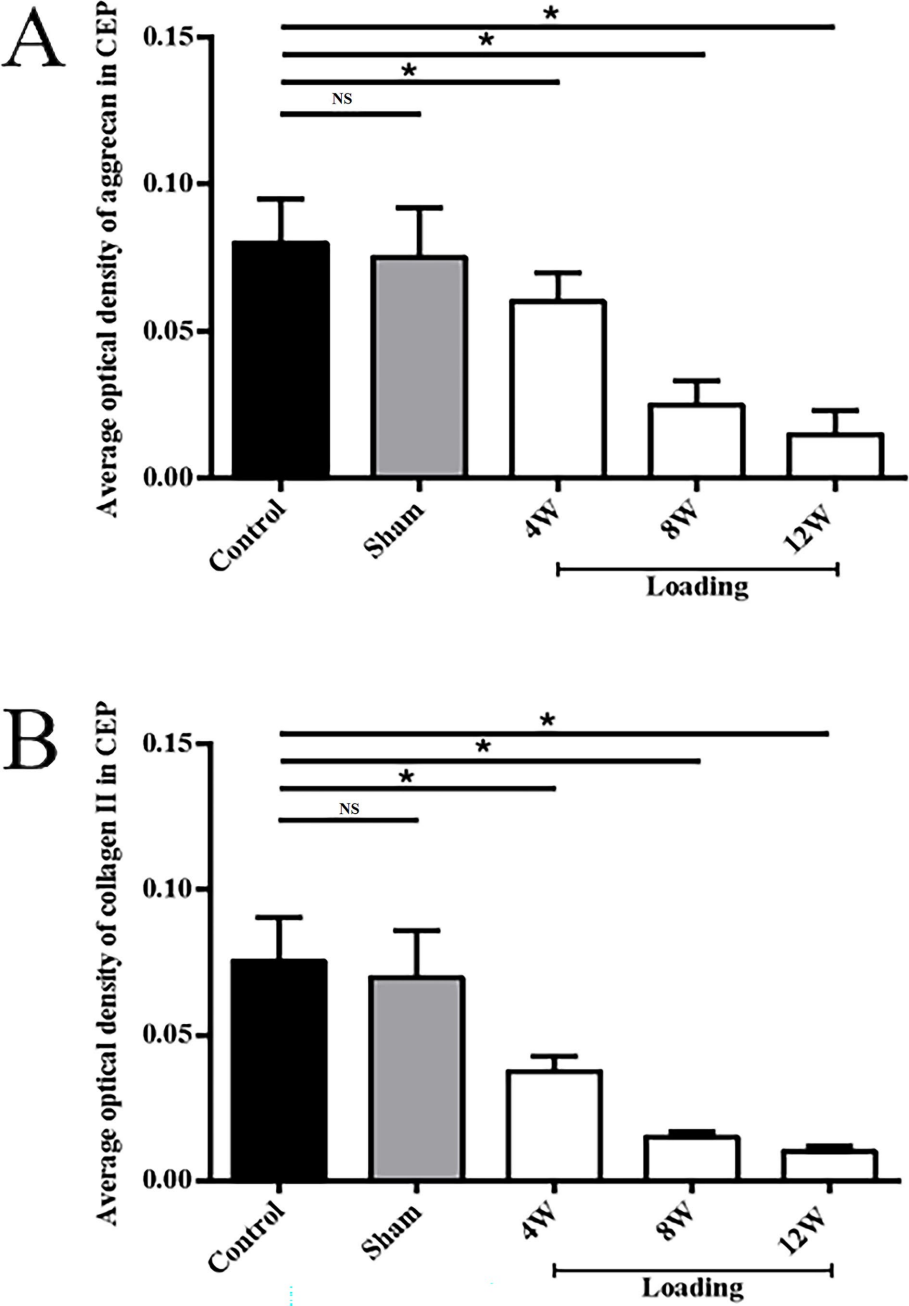
Comparisons of the average optical density values of Aggrecan and Collagen II in CEP and nucleus pulposus extracellular matrix (*p < 0.05; **p > 0.05). **(A)** the average optical density values of Aggrecan; **(B)** the average optical density values of Collagen II.

No significant differences in Aggrecan expression were observed between the control and sham groups for CEP (see Figures 5A,B, 6A, *p* = 0.947). However, following loading, a substantial reduction in Aggrecan expression was observed in the CEP. After 4 and 8 weeks of compression, the tissue structure integrity deteriorated significantly, resulting in minimal Aggrecan expression in enlarged CEP cells (Figures 5C–E). Statistical analysis of Aggrecan expression in CEP and nucleus pulposus revealed significant differences (*p* < 0.05) between the control and loading groups at each time point, as illustrated in Figure 6A.

In the control and sham groups, Collagen II content in both CEP and nucleus pulposus was abundant and concentrated (as depicted in Figures 5A, B, 6B), with no statistically significant differences in expression between the two groups (*p* = 0.863). Following 4–8 weeks of loading, the matrix exhibited gradual fragmentation and dispersion, accompanied by a significant decrease in nucleus pulposus content, while changes in CEP were less pronounced. Fibroblast-like cells were observed in the nucleus pulposus region (see Figures 5C,D). After 12 weeks of loading, positive staining for Collagen II was markedly reduced in both nucleus pulposus and CEP structures (see Figure 5E). Statistical analysis of matrix Collagen II expression in CEP and nucleus pulposus revealed significant differences (*p* < 0.05) between the control and loading groups at each time point, as illustrated in Figure 6B.

## 4 Discussion

While the nucleus pulposus has been extensively studied, the CEP tissue, despite its crucial role in the intervertebral disc function, has received comparatively less attention (20). However, the structural failure always started in CEP, indicating that these thin cartilage layers are the weak parts susceptible to excessive loading (21, 22). In this study, we aimed to induce CEP degeneration by developing a novel diurnal-axial loading device based on the Ilizarov-type apparatus and applying it *in vivo* to rat tails.

This device was designed to administer a stable and precise axial loading force. The compressive unit generated the loading force, propelling the motion of two aluminum rings along the rat tail’s caudal region. Kirschner wires, inserted through the aluminum rings and penetrating two adjacent vertebrae, were then brought into proximity. Subsequently, the targeted the intervertebral disc underwent compression with a pre-set force that could be meticulously adjusted under monitoring. Additionally, to assess the penetrating effects of the wires on the vertebrae and their potential impact on the CEP tissue, a sham operational group was established and examined. No significant signs of CEP degeneration were observed in the sham group, indicating negligible impact from wire penetration on the onset of CEP degeneration. Some loading devices have been constructed for inducing CEPD in the literature. One rat tail bending model was established by Lindblom et al. (23). They observed significant degeneration of the cartilage on the concave curved side, characterized by direct tissue structure damage and reduced cell numbers. Moreover, the degree of degeneration was found to be closely associated with both the duration of exposure and the magnitude of the bending force applied to the rat tail. Another classic Ilizarov-type compressive model which was widely used by many researchers due to its reliable installation and satisfactory modeling results (15, 24–26). However, these studies primarily used a set of springs to provide compression loads. Due to spring fatigue, a stable and reliable compressive effect could not be sustained over extended periods. Furthermore, the continuous static loading mode fails to replicate the physiological mechanical loading conditions experienced by the CEP. Therefore, we constructed a model providing diurnal load patterns in this study.

In our study, following loading, the targeted Co8/9 disc showed progressive degeneration over 4, 8, and 12 weeks, as evidenced by histological staining scores. The loaded disc displayed signs of severe and significant degeneration: micro-porosities in the CEP issue disappeared during the degeneration phase, and the nucleus pulposus underwent severe dehydration and fibrosis, accompanied by a notable decrease in the number of intervertebral cells. The degenerated discs ruptured through the compromised CEP, leading to the formation of Schmorl’s nodules. In the literature, Kuga et al. (27) found that in a fatigue stress test of spinal functional units, the annulus fibrosus is prone to rupture, and the CEP is a weak area vulnerable to repetitive motion stress. Zehra et al. (28) investigated that abnormal pressure loading can lead to CEP failure, showing that the CEP thins with degeneration. They also observed a strong correlation between the intervertebral disc degenerationand multiple, large CEP defects, which may affect the volume and compression within the nucleus pulposus (29). Maclean et al. (30) observed the effects of short-term high-force compressive loading on rat intervertebral discs using an Ilizarov-type device. Their findings indicated that abnormal compression can result in reduced expression of collagen I and II and increased expression of catabolic genes such as Collagenase-3, potentially leading to the breakdown of Collagen II, a critical extracellular matrix component in the intervertebral disc.

As degeneration progresses, Collagen II gradually diminishes from the extracellular matrix. Immunohistochemical analysis revealed a significant reduction in Collagen II expression in the nucleus pulposus and CEP of the experimental group compared to the control group. Specifically, at 4, 8, and 12 weeks post-operation, Collagen II expression decreased by 39.8, 65.4, and 80.7% in the nucleus pulposus and by 22.5, 56.2, and 71.3% in the CEP, respectively. Similarly, Collagen II expression decreased by 42.6, 57.9, and 73.4% in the nucleus pulposus and by 48.6, 74.8, and 86.5% in the CEP, respectively. No significant difference was observed in Aggrecan and Collagen II expression in the Co8/9 disc between the sham operation group and the control group (*p* = 0.36), suggesting that nutritional disorder due to the modeling procedure may not have significantly influenced the results. High-force loading was found to disrupt intracellular environments, activate inflammatory factors, and induce matrix metalloproteinase production, ultimately leading to continuous extracellular matrix decomposition and destruction. Yamazaki et al. (31) proposed that abnormal loading would up-regulate the catabolism related factors and induce the expression increase of matrix degrading and proteolytic enzyme. Ariga et al. (32)conducted an *ex-vivo* study and observed increased number of apoptotic CEP cells with increased static mechanical loading force.

There are several limitations in the current study. First, this model only considers axial compression load, yet the biomechanics of the CEP are far more complex. To date, there is no *in vivo* study that comprehensively simulates the effects of various mechanical forces, including loading, torsion, and shear force, on the CEP (33). Second, the choice of rats as the experimental model presents challenges in directly comparing results with human discs. Compared to larger animals, such as goats, cattle, or even apes, rat intervertebral discs have higher water content in their extracellular matrix and contain notochordal cells, which are absent in adult human discs. Future research directions should focus on developing a more sophisticated compressive device with adjustable frequencies and loading settings that can be controlled wirelessly. This would allow for more precise and dynamic manipulation of mechanical forces applied to the CEP. Furthermore, consideration should be given to employing large animal models with biomechanical characteristics more closely resembling those of humans, such as goats or apes. These models could provide more translatable insights into CEP degeneration processes and potential therapeutic interventions.

## 5 Conclusion

Excessive axial loading induced CEPD in the rat tail, primarily characterized by the formation of Schmorl’s nodes and a reduction in CEP microporosity in this study. Our modified Ilizarov rat tail compression model, featuring stable and controllable axial loading capabilities, provided an alternative experimental paradigm for further investigation into CEPD.

## Data Availability

The original contributions presented in the study are included in the article/Supplementary material, further inquiries can be directed to the corresponding author.
