# Supplementary figures and images for: What can be observed in intervertebral cartilage endplate with aging? An animal model study of excessive axial mechanical loading
Source: Front Med (Lausanne). 2024 Nov 5;11:1429208. doi: 10.3389/fmed.2024.1429208 (PMC11573515; doi:10.3389/fmed.2024.1429208)

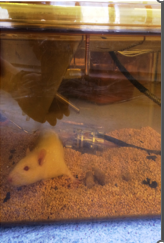

Supplement: Supplementary file 1 [file Image_1.PNG]
